# SLC25 Family Member Genetic Interactions Identify a Role for *HEM25* in Yeast Electron Transport Chain Stability

**DOI:** 10.1534/g3.117.041194

**Published:** 2017-04-12

**Authors:** J. Noelia Dufay, J. Pedro Fernández-Murray, Christopher R. McMaster

**Affiliations:** * Department of Biochemistry and Molecular Biology, Dalhousie University, Halifax, Nova Scotia B3N 0A1, Canada; † Department of Pharmacology, Dalhousie University, Halifax, Nova Scotia B3N 0A1, Canada

**Keywords:** heme, mitochondria, electron transport chain, SLC25 protein family, glycine import

## Abstract

The SLC25 family member SLC25A38 (Hem25 in yeast) was recently identified as a mitochondrial glycine transporter that provides substrate to initiate heme/hemoglobin synthesis. Mutations in the human *SLC25A38* gene cause congenital sideroblastic anemia. The full extent to which SLC25 family members coregulate heme synthesis with other mitochondrial functions is not clear. In this study, we surveyed 29 nonessential SLC25 family members in *Saccharomyces cerevisiae* for their ability to support growth in the presence and absence of *HEM25*. Six SLC25 family members were identified that were required for growth or for heme synthesis in cells lacking Hem25 function. Importantly, we determined that loss of function of the SLC25 family member Flx1, which imports FAD into mitochondria, together with loss of function of Hem25, resulted in inability to grow on media that required yeast cells to supply energy using mitochondrial respiration. We report that specific components of complexes of the electron transport chain are decreased in the absence of Flx1 and Hem25 function. In addition, we show that mitochondria from *flx1*Δ *hem25*Δ cells contain uncharacterized Cox2-containing high molecular weight aggregates. The functions of Flx1 and Hem25 provide a facile explanation for the decrease in heme level, and in specific electron transport chain complex components.

Heme is a component of many cellular constituents including respiratory cytochromes, P450 cytochromes, catalase, peroxidase, myoglobin, and hemoglobin (Ajioka *et al.* 2006; Chiabrando *et al.* 2014; Yuan *et al.* 2013; Kardon *et al.* 2015). Heme biosynthesis is catalyzed by eight enzymes located in the cytoplasm and mitochondria (Figure 1A). The first enzymatic reaction in heme synthesis takes place in mitochondria through the condensation of glycine with succinyl-CoA to form 5-aminolevulinic acid (5-Ala) (Aivado *et al.* 2006; Bishop *et al.* 2012). In the yeast *Saccharomyces cerevisiae* this reaction is catalyzed by Hem1, while in humans it is catalyzed by two different aminolevulinic acid synthases (ALAS), one expressed ubiquitously (ALAS1) and the other expressed in erythroid precursor cells (ALAS2) (Ajioka *et al.* 2006; Chiabrando *et al.* 2014; Yuan *et al.* 2013; Fernández-Murray *et al.* 2016). ALAS requires the cofactor pyridoxal 5-phosphate (PLP) to catalyze its reaction (Astner *et al.* 2005). 5-Ala is exported to the cytoplasm, where, through a sequence of enzymatic reactions (Figure 1A), coproporphyrinogen III is synthesized. Coproporphyrinogen III is imported from the cytoplasm into mitochondria, where it is converted through a sequence of enzymatic reactions into heme (Figure 1A) (Chiabrando *et al.* 2014). Heme is subsequently incorporated into mitochondrial proteins, including those within respiratory cytochromes of the electron transport chain (ETC) and mitochondrial P450 enzymes. Heme is also transported across the mitochondrial membrane into the cytoplasm, where heme chaperons bind and transport heme for incorporation into various hemoproteins, including microsomal P450 enzymes and other detoxifying enzymes, and in erythroid cells with globin chains to form hemoglobin (Weatherall 2013).

**Figure 1:**
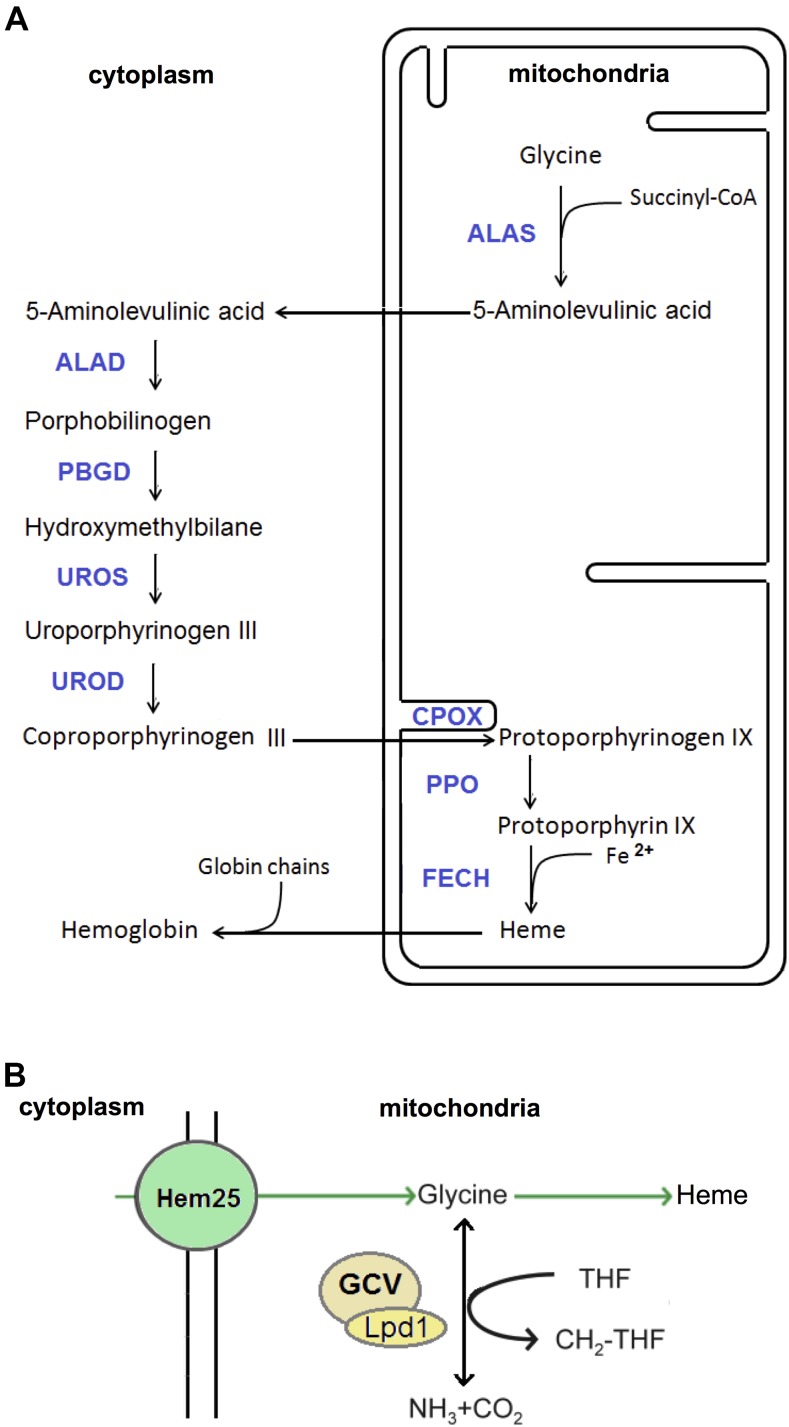
Mitochondrial glycine is used to synthesize heme and produce one-carbon units. (A) Heme biosynthesis pathway. (B) Mitochondrial glycine can be used to synthesize heme or catabolized by the GCV into nitrogen and one-carbon units. Hem25, the yeast homolog of human SLC25A38, is a mitochondrial glycine importer, and Lpd1 is a subunit of the yeast GCV. ALAS, aminolevulinic acid synthase; ALAD, aminolevulinic acid dehydratase; PBGD, porphobilinogen deaminase; UROS, uropoporphyrinogen III synthase; UROD, uroporphyrinogen III decarboxylase; CPOX, coproporphyrinogen oxidase; PPO, protoporphyrinogen oxidase; FECH, ferrochelatase; GCV, glycine cleavage complex.

Mitochondria possess both outer and inner membranes with differential permeabilities. The outer mitochondrial membrane is permeable to solutes up to ∼5 kDa, while the inner membrane is comparatively impermeable to enable efficient oxidative phosphorylation. Various transporters reside within the inner mitochondrial membrane to overcome its permeability barrier, including transporters required to facilitate the synthesis of heme. The identities of the transporters required for heme biosynthesis are still being unraveled. The exporter of 5-Ala out of the mitochondria has been hypothesized to be ABCB10 (Chen *et al.* 2009, 2010; Shintre *et al.* 2013; Tang *et al.* 2012; Yamamoto *et al.* 2014)—a member of the ABC transporter family that has an important role during erythroid differentiation, and whose over expression promotes hemoglobin synthesis. The mitochondrial importer of coproporphyrinogen III has been proposed to be ABCB6, which binds both porphyrins and heme; moreover, ABCB6 expression is positively regulated by the stimulation of erythroid differentiation and by heme levels (Paterson *et al.* 2007; Zhang *et al.* 2013; Zhao *et al.* 2013; Krishnamurthy *et al.* 2006; Schultz *et al.* 2010). Several lines of evidence point to the mitochondrial heme exporter being FLVCR1b (Chiabrando *et al.* 2012; Fleming and Hamza 2012). FLVCR1b is essential for erythroid differentiation, and overexpression of FLVCR1b promotes heme biosynthesis, whereas its silencing results in mitochondrial heme accumulation. In addition, FLVCR1a is a heme exporter that resides in the plasma membrane (Chiabrando *et al.* 2012). Other mitochondrial membrane transporters have recently been implicated in the synthesis of heme, in particular, members of the SLC25 protein family (Gutierrez-Aguilar and Baines 2013).

The SLC25 transporter family member SLC25A38 was recently identified as a glycine transporter that provides substrate for ALAS2 to initiate heme/hemoglobin synthesis in the mitochondria of erythropoietic cells (Fernández-Murray *et al.* 2016; Lunetti *et al.* 2016). Another SLC25 family member, SLC25A32, imports folate into mitochondria (Urano *et al.* 2014; Lawrence *et al.* 2011). SLC25A32 appears to be essential for glycine synthesis inside mitochondria as folate is required for the conversion of serine to glycine by mitochondrial serine hydroxymethyltransferase (Locasale 2013; Giardina *et al.* 2015; Saint-Marc *et al.* 2015). Thus, both the import of glycine into the mitochondria, and glycine synthesis by mitochondria, require SLC25 family members. Glycine is required for the first step in heme synthesis, as well as other mitochondrial processes, including its use as a substrate for the glycine cleavage complex (GCV) (Tibbetts and Appling 2010; Wang *et al.* 2013). Mitochondrial folate is also required for the GCV, which produces one-carbon units required for the synthesis of several macromolecules, including formylmethionine for mitochondrial translation initiation (Tibbetts and Appling 2010; Wang *et al.* 2013). It has also been suggested that SLC25A32 could transport FAD across the inner mitochondrial membrane (Spaan *et al.* 2005; Gutierrez-Aguilar and Baines 2013). Other SLC25 transporters, including SLC25A28 and SLC25A37 (also referred to as mitoferrin-2 and mitoferrin-1, respectively), contribute to heme metabolism while also contributing to other mitochondrial functions (Chen *et al.* 2009; Urano *et al.* 2014). SLC25A28 and SLC25A37 both transport iron into mitochondria. Iron is required for the last step of heme biosynthesis, and for the formation of Fe–S clusters. Fe–S clusters are incorporated into many mitochondrial enzymes, including several within the ETC (Ajioka *et al.* 2006; Gomez *et al.* 2014; Wingert *et al.* 2005). The SLC25 family members SLC25A4, SLC25A5, SLC25A6, and SLC25A31 are adenine nucleotide translocases that are the main transporters of ADP into the mitochondria and ATP to the cytoplasm; however, they have also been proposed to transport heme or heme precursors across mitochondrial membranes (Azuma *et al.* 2008; Fleming and Hamza 2012; Yuan *et al.* 2013). It is clear that SLC25 family members are important for the synthesis of heme while simultaneously regulating other mitochondrial processes.

The extent to which the mitochondrial SLC25 family members regulate heme synthesis is not known. In this study, new SLC25 family members whose function is required to maintain normal heme level were identified and characterized. In addition, we describe a previously unknown SLC25 family mediated relationship that simultaneously regulates heme synthesis and ETC function.

## Materials and Methods

### Yeast strains

Yeast strains used are listed in Supplemental Material, Table S1 in File S1. In some instances, the *KanMX4* gene, which had been used to inactivate yeast genes obtained from the yeast deletion collection, was replaced with the nourseothricin acetyltransferase (*NatMX4*) gene. This was done by transformation of the corresponding strain with a linearized pAG25 plasmid as described (Voth *et al.* 2003). Briefly, the linearized plasmid was transformed into yeast cells, cells were plated and grown in yeast peptone dextrose (YPD) medium for 1 d, and were replica plated onto YPD medium containing nourseothricin. Antibiotic resistant colonies were restreaked to isolate single-cell-derived colonies. Proper antibiotic resistance gene replacement was assessed by the consequent loss of G-418 resistance character. BY4741 and BY4742 strains carry a Ty1 element inserted in the 3′ region of the *HAP1* open reading frame (Buschlen *et al.* 2003; Gaisne *et al.* 1999). The *HAP1* gene encodes a transcriptional regulator involved in regulation of gene expression in response to heme and oxygen levels, thus strains from the BY4741 and BY4742 backgrounds used in this study were transformed with a low-copy plasmid carrying a wild-type allele of *HAP1* and a selectable *URA3* marker.

To study the genetic interactions of 29 nonessential yeast SLC25 family members with the SLC25 family member *HEM25*, double gene deletion strains were constructed by standard genetic crosses of *hem25*Δ haploid cells with each SLC25 family member single gene deletion strain. Following diploid cell section, sporulation, and haploid cell selection, single colonies were isolated that contained a deletion of the *HEM25* gene with each yeast SLC25 family member. During the process of construction of the double mutant strains, the media used were supplemented with 5 mM glycine and 0.38 mM 5-Ala.

### Growth assays

Cells were grown overnight in liquid synthetic dextrose (SD) medium without uracil (SD–URA) containing 1 g/liter of ammonium sulfate. Cells were washed twice, and resuspended in liquid SD–URA medium containing 30 g/liter glycine as the sole nitrogen source. An OD_600 nm_ 0.1 was standardized for every culture at time 0, cells were grown at 30°, and OD_600 nm_ was monitored to determine growth rate.

The growth of yeast strains on plates was estimated using a serial dilution assay. Cells were grown to late log phase (0.9–1 OD_600 nm_) at 30° in SD–URA, the cell density of the culture was adjusted to OD_600_ 0.4, serially diluted 1:10 four times, spotted onto appropriate solid medium using a replica pinner, and incubated at 30°. Plates were imaged using a Bio-Rad VersaDoc.

### Isolation of mitochondria

Mitochondrial fractions were prepared by differential centrifugation as previously described (Diekert *et al.* 2001; Gaspard and McMaster 2015). Briefly, yeast strains were grown at 30° until they reached midlog phase. Cells were washed and inoculated in 200 ml of synthetic medium without uracil with raffinose as the carbon source at OD_600 nm_ 0.2, and grown until OD_600 nm_ 1–1.5. Cells were washed and transferred to synthetic medium without uracil with lactate as the carbon source for 5 hr. For mitochondrial samples to be analyzed by SDS-PAGE, cells were collected by centrifugation at 2500 × *g* for 5 min at 4°, and the cell pellet was resuspended in ice-cold 0.6 M sorbitol, 20 mM HEPES-KOH, 1 mM phenylmethanesulfonylfluoride at a density of 0.5 g cells/ml. Cells were lysed by vortexing twice for 15 sec at maximum speed in the presence of glass beads at 4°. Unbroken cells, cell debris and nuclei were spun down at 600 × *g* for 5 min at 4°. The supernatant was carefully removed from the pellet and centrifuged for 10 min at 10,000 × *g* at 4°. The resulting crude mitochondrial pellet was resuspended in 20 μl of 50 mM Tris-HCl, pH 7.0, 1% sodium dodecyl sulfate (SDS). Then, 10 μl of 3% SDS, 132 mM Na_2_CO_3_, 4% β-mercaptoethanol was added to the mitochondrial suspension followed by urea to a 6 M final concentration. This crude mitochondrial preparation, subjected to SDS-PAGE and western blot analysis, was used to estimate the relative abundance of several mitochondrial proteins.

To assess the integrity of mitochondrial respiratory supercomplexes by Blue Native (BN)-PAGE, mitochondria were isolated by differential centrifugation (Diekert *et al.* 2001; Gaspard and McMaster 2015) Briefly, harvested cells were washed once in distilled water, resuspended in 30 ml of freshly prepared TD buffer (100 mM Tris-sulfate, pH 9.4, 10 mM DTT) and incubated for 5 min at 30° with gentle shaking. These cells were then collected by centrifugation at 2500 × *g* for 5 min and resuspended in SP buffer (1.2 M sorbitol, 20 mM potassium phosphate, pH 7.4). Spheroplasts were generated by treatment with zymolase 100T (1.5 mg/g cells) for 30–60 min at 30° with gentle shaking. Spheroplasts were harvested by centrifugation at 2500 × *g* for 5 min at 4°, and then washed twice with 40 ml of ice-cold SP buffer. After the washes, spheroplasts were resuspended in 60 ml of ice-cold SH buffer (0.6 M sorbitol, 20 mM HEPES-KOH, pH 7.4) with 1 mM phenylmethanesulfonylfluoride and complete protease inhibitors (Roche). The suspension was transferred to a large piston glass homogenizer (Potter-Elvehjem-Type Tissue Grinders), and homogenized with 25 strokes; the homogenized mixture was then subjected to centrifugation twice at 2500 × *g* for 5 min at 4°. The supernatants were pooled together in a fresh 50 ml glass tube, and pelleted by centrifugation at 12,000 × *g* for 10 min at 4°. Using a small-scale glass homogenizer, the pellet was first carefully resuspended in 1 ml of SH buffer, and then resuspended in 25 ml of ice cold SH buffer. This suspension was further centrifuged at 2500 × *g* for 5 min at 4°, the supernatant was saved and then pelleted at 12,000 × *g* for 10 min at 4°. The resulting pellet (mitochondria) was resuspended in 0.5 ml of SH buffer and divided into aliquots containing 0.1–0.3 mg protein (estimated by a modified Lowry method; Markwell *et al.* 1978). The aliquots were flash-frozen in liquid nitrogen and stored at −70° until further analysis.

### BN-PAGE

The status of yeast respiratory chain supercomplexes was assessed by BN-PAGE as described previously with minor modifications (Wittig *et al.* 2006). Frozen mitochondria (150 μg of protein) were thawed, collected by centrifugation at 12,000 × *g* for 10 min, and suspended in 15 μl of 50 mM NaC1, 2 mM 6-aminohexanoic acid, 50 mM imidazole/HC1, pH 7.0. To solubilize membranes, 6 μl of 5% digitonin (detergent/protein ratio of 2 g/g; Invitrogen) was added to this mixture, incubated for 10 min on ice, and centrifuged at 20,000 × *g* for 20 min at 4°. Alternatively, mitochondrial proteins were solubilized with dodecylmaltoside (detergent/protein ratio of 1 g/g). The supernatant containing the solubilized mitochondrial proteins was transferred to a new tube, and 2 μl of 50% glycerol and 0.8 μl of 5% Coomassie blue G-250 (Pierce) were added. Solubilized mitochondrial proteins were separated on a 3–12% precast Bis-Tris Native PAGE (Invitrogen) using a XCell sure lock Mini-Cell gel running tank (Invitrogen) by the application of constant current at 150 V for 3 hr in a cold room. To improve detection of faint protein bands and to maximize protein transfer to PVDF membranes, the dark cathode buffer with 0.02% Coomassie blue G-250 was replaced with light cathode buffer (0.002% Coomassie blue G-250) once the proteins reached one-third of the running distance in BN-PAGE. A diluted aliquot of ferritin, which runs as two visible (brown) bands with molecular masses of 440 and 880 kDa was separated along with the samples by BN-PAGE to serve as a molecular marker. For western blotting, the BN gel was soaked for 10 min in electrode buffer (50 mM tricine, 7.5 mM imidazole, pH 7.0) containing 1% SDS and the proteins were then transferred to PVDF membranes using a semidry electroblotting apparatus in the presence of electrode buffer at 4° for 3 hr under limiting current (0.5 mA/cm^2^ of gel area) and voltage set at 20 V. Supercomplexes containing complex IV were probed with a monoclonal anti-Cox2 antibody (1:1000; from MitoSciences) in the presence of 0.02% SDS. Complex V, detected with a polyclonal anti-F1α antibody (1:1000; Dr. C. Koehler), was used as loading control. Primary antibodies were detected with HRP-conjugated secondary antibodies (from Cell Signaling), followed by chemoluminescense.

### Western blot analysis

Protein concentration was determined by a modified Lowry method (Markwell *et al.* 1978). This modified method utilizes the detergent deoxycholate to solubilize hydrophobic proteins present in membranes. The mitochondrial fractions were added to loading buffer (0.05% bromophenol blue, 25% glycerol, 6% SDS, 6 mM EDTA, and 150 mM Tris-HCl pH 8.8 and 0.5% β-mercaptoethanol), and incubated for 1 hr at 37°. Samples were resolved by 12% SDS-PAGE and the separated proteins were transferred to a nitrocellulose membrane. The membranes were incubated for 1 hr under constant shaking in Odyssey blocking buffer (LI-COR). The blots were then incubated with an appropriate dilution of primary antibody (Table S2 in File S1) in blocking buffer under constant shaking overnight at 4°. The following day, the membranes were washed three times for 10 min each with 0.1% Tween-20 in phosphate-buffered saline (PBS), and were then incubated with an appropriate dilution of secondary antibody in LI-COR blocking buffer for 1 hr. Excess antibody was washed off with 0.1% Tween-20 in PBS and proteins were detected and imaged by using a LI-COR Odyssey Infrared Imaging System. The membranes were stripped with New Blot Nitro Stripping Buffer (LI-COR) for 10 min under constant shaking at room temperature, and reprobed with anti-porin antisera (Por1) as a loading control.

### Heme determination

Logarithmically growing cells in SD-Ura media were harvested, washed in ice-cold water, and resuspended in 10 mM Tris-HCl (pH 8.0), 150 mM NaCl. Cells were lysed by vortexing with glass beads for two periods of 1 min intercalated with 1 min on ice. Cell debris was removed by centrifugation at 500 × *g* for 3 min, and heme and protein content of the supernatant were assayed. Heme determination was carried out using the Hemin Assay Kit from BioVision (Atamna *et al.* 2015).

### Data availability

The authors state that all data necessary for confirming the conclusions presented in the article are represented fully within the article.

## Results

### Growth impairment by simultaneous deletion of HEM25 and members of the SLC25 family

The ability of a yeast cell to utilize glycine as a nitrogen source depends on the transport of glycine inside the mitochondria, which is mediated mostly by Hem25 followed by the catabolism of glycine into ammonia through the GCV (Figure 1B). We generated 29 double deletion yeast strains, whereby the *HEM25* gene was inactivated in cells lacking each nonessential SLC25 family member. We hypothesized that inactivation of these SLC25 family members could affect the utilization of glycine as a nitrogen source. We anticipated that the absence of any other member of the SLC25 family that contributed to glycine import, or toward the functionality of the GCV, would exacerbate the growth defect of a *hem25*Δ strain. For comparison, an *lpd1*Δ strain, defective in lipoamide dehydrogenase that renders the GCV inactive, and a wild-type strain, were included.

**Figure 2:**
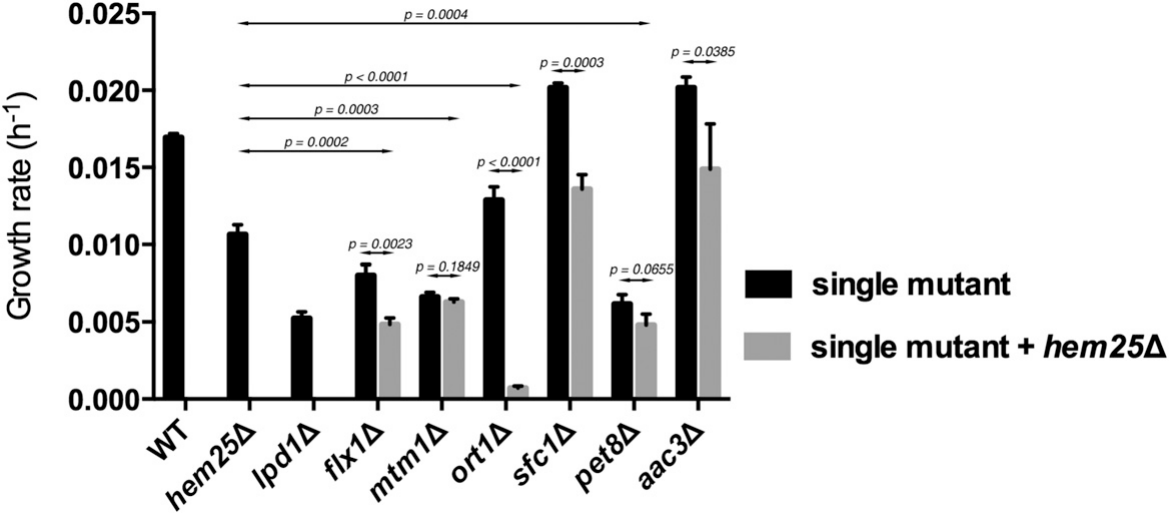
Growth rates on glycine as sole nitrogen source for selected members of the SLC25 family in combination with *HEM25* deficiency. Cells of the indicated genotypes were grown overnight on SD-Ura medium containing 1 g/liter of ammonium sulfate at 30°, washed twice and inoculated at OD_600 nm_ 0.1 in SD-Ura medium containing 30 g/liter of glycine. Cells were cultivated at 30°, and growth was monitored spectrophotometrically. Differences between single deletion strains and double deletion strains were determined using ANOVA test with randomized factors. At least three independent experiments were done to calculate the p values, mean, and SEM. The numbers represented in the graph are growth rates calculated over a period of 4 d.

*FLX1*, *MTM1*, *ORT1*, *SFC1*, *PET8*, and *AAC3* genes were selected based on the growth phenotype on glycine as a nitrogen source of the null mutants alone or in combination with inactivated *HEM25* (Figure S1 in File S1 and Table 1). To better assess the possible synthetic interaction between these genes and *HEM25*, we determined their growth rates on glycine as nitrogen source (Figure 2). The *ort1*Δ *hem25*Δ showed a synthetic growth defect. Double mutant strain *flx1*Δ *hem25*Δ grew poorly compared to wild-type cells and to the single gene knockout strains *hem25*Δ and *flx1*Δ, with the growth impairment of the double mutant reflecting the cumulative effect of each single mutant. Single mutant strains *mtm1*Δ and *pet8*Δ showed a severe growth phenotype that was not exacerbated when *HEM25* was inactivated. Interestingly, *sfc1*Δ and *aac3*Δ cells grew faster than wild type when glycine was the nitrogen source; however, this growth improvement was reduced proportionately by the loss of *HEM25*.

**Table 1:** Identified and known functions of SLC25 family members that genetically interact with *HEM25*

Standard Name	Systematic Name	Known Function(s)
*FLX1*	*YIL134w*	Mitochondrial flavin adenine dinucleotide transporter; FAD is a synthesis product of riboflavin; human homolog *SLC25A32* is implicated in multiple acyl-CoA dehydrogenase deficiency (MADD) or glutaric aciduria type II (GAII), and can complement yeast null mutant
*MTM1*	*YGR257c*	Mitochondrial protein of the mitochondrial carrier family; high affinity pyridoxal 5′-phosphate (PLP) transporter, important for delivery of PLP cofactor to mitochondrial enzymes; involved in mitochondrial iron homeostasis
*ORT1*	*YOR130c*	Ornithine transporter of the mitochondrial inner membrane; exports ornithine from mitochondria as part of arginine biosynthesis; functionally complemented by human ortholog, *SLC25A15*, which is associated with hyperammonaemia-hyperornithinaemia-homocitrullinuria (HHH) syndrome, but HHH-associated variants fail to complement
*SFC1*	*YJR095w*	Mitochondrial succinate-fumarate transporter; transports succinate into and fumarate out of mitochondria; required for ethanol and acetate utilization
*PET8*	*YNL003c*	S-adenosylmethionine transporter of the mitochondrial inner membrane; member of the mitochondrial carrier family; required for biotin biosynthesis and respiratory growth
*AAC3*	*YBR085w*	Mitochondrial inner membrane ADP/ATP translocator; exchanges cytosolic ADP for mitochondrially synthesized ATP; expressed under anaerobic conditions; has roles in maintenance of viability and in respiration

### Heme content was decreased in cells lacking SLC25 family members

**Figure 3:**
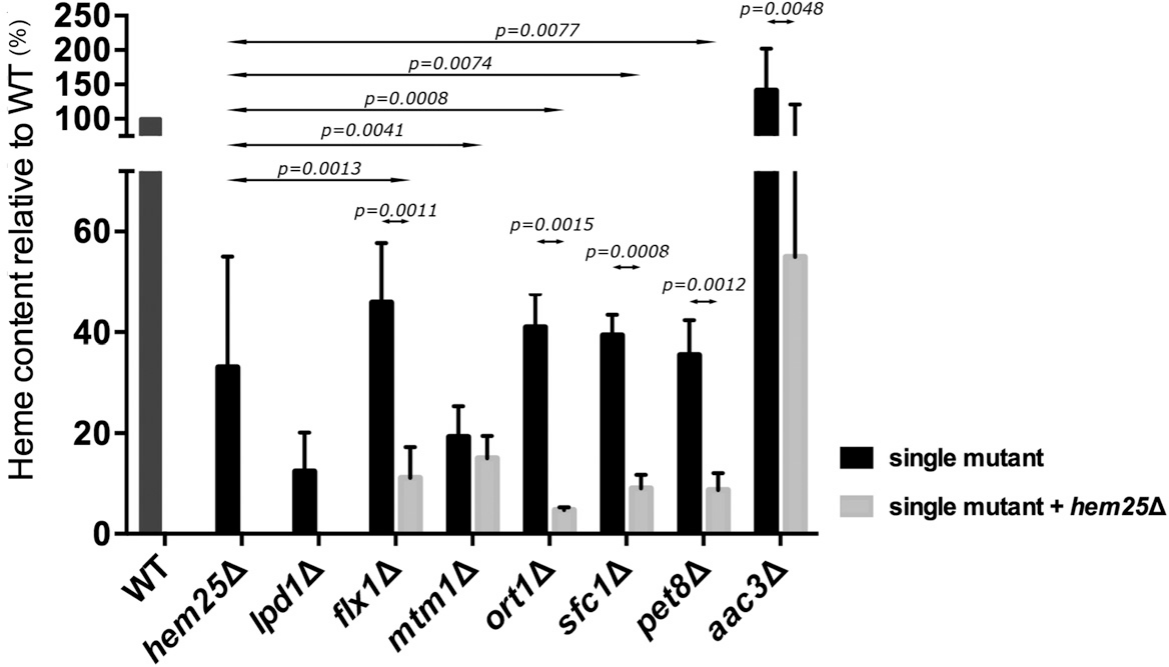
Heme content for selected members of the SLC25 family in combination with *HEM25* deficiency. Cells of the indicated genotypes were grown at OD_600 nm_ 0.6–1 in SD-Ura. Cells were harvested and processed for heme determination. Differences between single deletion strains and double deletion strains were determined using ANOVA test with randomized factors. At least three independent experiments were done to calculate the p values, mean, and SEM. Values are normalized to wild type (WT). WT heme level was 28.9 ± 4.2 fmol/μg of protein.

In mitochondria, glycine is a substrate for the synthesis of several molecules, including heme (Fernández-Murray *et al.* 2016; Schonauer *et al.* 2009; Tibbetts and Appling 2010; Wang *et al.* 2013). We determined if inactivation of *FLX1*, *MTM1*, *ORT1*, *SFC1*, *PET8*, and *AAC3* genes, *per se* or in combination with ablation of *HEM25* gene, altered heme level. Heme levels were compared to the heme level found in wild type, *hem25*Δ, and *lpd1*Δ cells. Lpd1 is a subunit of both the GCV and the KGD complex. The KGD complex catalyzes the synthesis of succinyl-CoA, which is used along with glycine to synthesize 5-Ala to initiate heme synthesis (Heublein *et al.* 2014; Schonauer *et al.* 2009; Ajioka *et al.* 2006; Yuan *et al.* 2013). The *hem25*Δ cells had a heme level 35% that of wild type, while *lpd1*Δ cells had a heme level 12% that of wild type (Figure 3). Similar to *hem25*Δ cells, there was a decrease in heme content in cells lacking *FLX1*, *ORT1*, *SFC1*, or *PET8*, to 38–49% of wild type, while cells lacking *MTM1* had heme levels that were 22% wild type (Figure 3). Interestingly, cells lacking *AAC3* had a heme content that was 150% that of wild type cells. Inactivation of the *HEM25* gene in each of these single mutants to create double mutant cells all resulted in a further decrease in heme content compared to the single mutant strains. The double *ort1*Δ *hem25*Δ exhibited a synthetic phenotype having its heme levels reduced beyond the cumulative effect of each single mutation (Figure 3), consistent with the observed growth defect for this double mutant when using glycine as nitrogen source. In the double mutant strains lacking *HEM25* and either *FLX1*, *SFC1*, or *PET8*, heme content was 12–17% that of wild type. The double mutant *aac3*Δ *hem25*Δ had a reduction in heme content to 57% that observed in wild type cells, which was significantly less than that observed in *aac3*Δ cells but was higher than the level observed in *hem25*Δ cells. When *MTM1* was deleted in *hem25*Δ cells, the heme content did not significantly decrease beyond the low heme level already present upon inactivation of just the *MTM1* gene.

### Glycine and 5-Ala restore growth to strains lacking selective SLC25 family members

**Figure 4:**
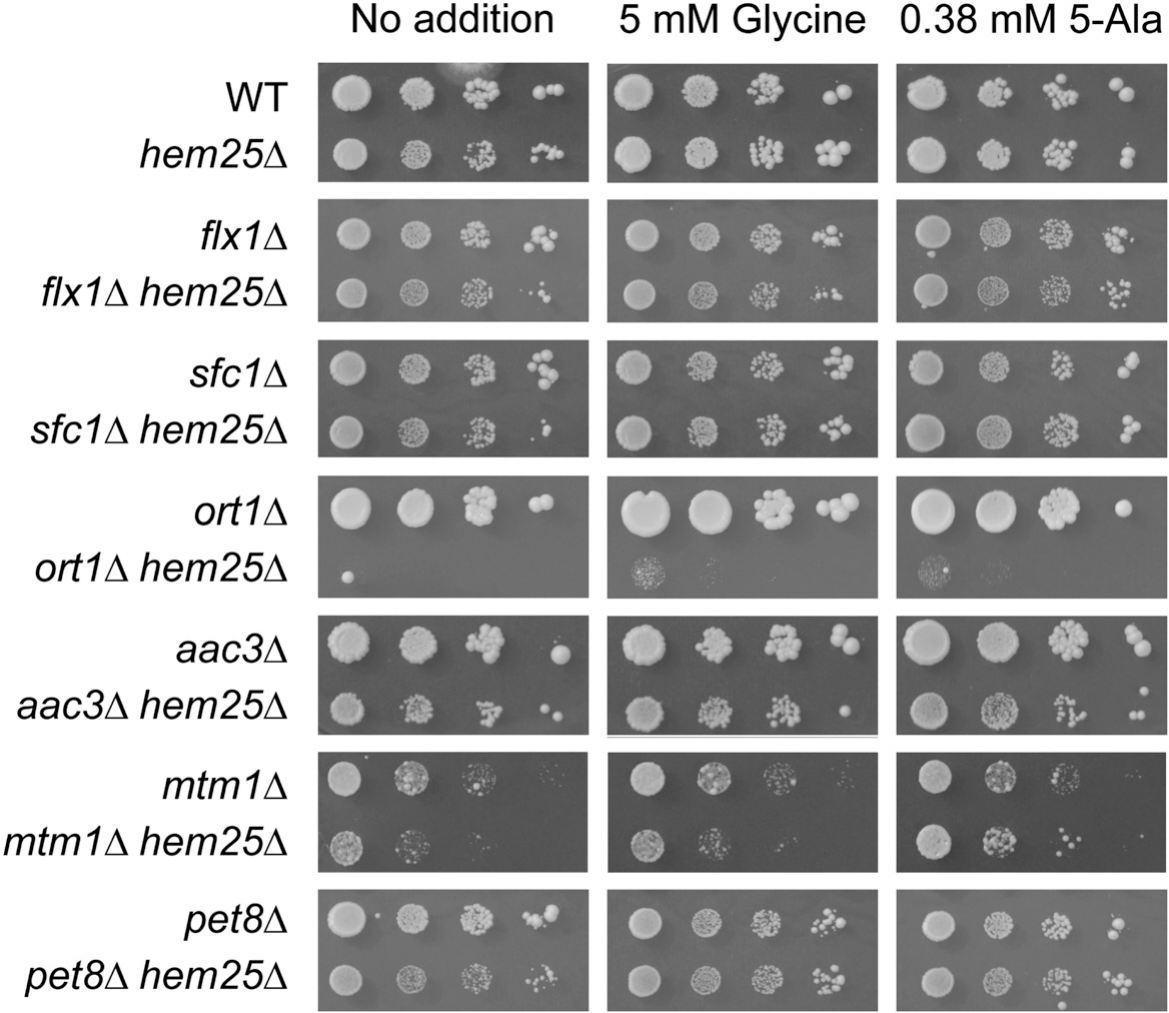
Glycine and 5-Ala alleviate the growth defect of a subset of SLC25 family members upon inactivation of the mitochondrial glycine importer *HEM25*. Yeast strains of the indicated genotypes were grown to early stationary phase in SC-Ura medium supplemented with 5 mM glycine and 0.38 mM 5-Ala to keep the double mutant cells growing without impediment. Cells were washed and resuspended in sterilized water to OD_600 nm_ 0.4, then serially diluted (1:10) and spotted on SD-Ura solid media containing dextrose as carbon source. Plates were imaged after 3 d incubation at 30°.

We sought to determine if the genes whose inactivation, *per se* or in combination with inactivated *HEM25* cells that resulted in impaired growth on glycine as nitrogen source and reduced heme content, had a role specific to heme synthesis, or if they participated with glycine in a mitochondrial function beyond heme synthesis. We assessed if growth was restored by the addition of excess glycine (5 mM), or by the heme-specific biosynthetic metabolite 5-Ala (0.38 mM) (Figure 4) (Fernández-Murray *et al.* 2016). To enable determination of the effect of glycine and 5-Ala, these experiments were carried out on yeast minimal medium containing dextrose as the carbon source and ammonium sulfate as the nitrogen source. Under normal growth conditions, loss of Hem25 function did not affect growth, compared to the 25% decrease in growth rate when glycine was used as the sole nitrogen source. In addition, in *hem25*Δ cells, inactivation of *AAC3*, *MTM1*, and *PET8* all slightly decreased growth beyond that of each single mutant, inactivation of *ORT1* almost completely abrogated growth, while inactivation of *FLX1* and *SFC1* did not substantially alter growth. Thus, unlike when glycine was used as the sole nitrogen source, a decrease in growth was observed when ammonium sulfate was used as the sole nitrogen source only for *hem25*Δ cells lacking *AAC3*, *MTM1*, *PET8*, and *ORT1*, but not for *FLX1* and *SFC1*. Interestingly, only the *hem25*Δ *mtm1*Δ strain growth defect was alleviated by the addition of 5-Ala, but not by glycine. The *hem25*Δ *pet8*Δ strain was the only one whose (slight) growth impairment was relieved by the addition of either 5-Ala or glycine. Glycine and 5-Ala supplementation did not affect the growth of any of the other strains.

**Figure 5:**
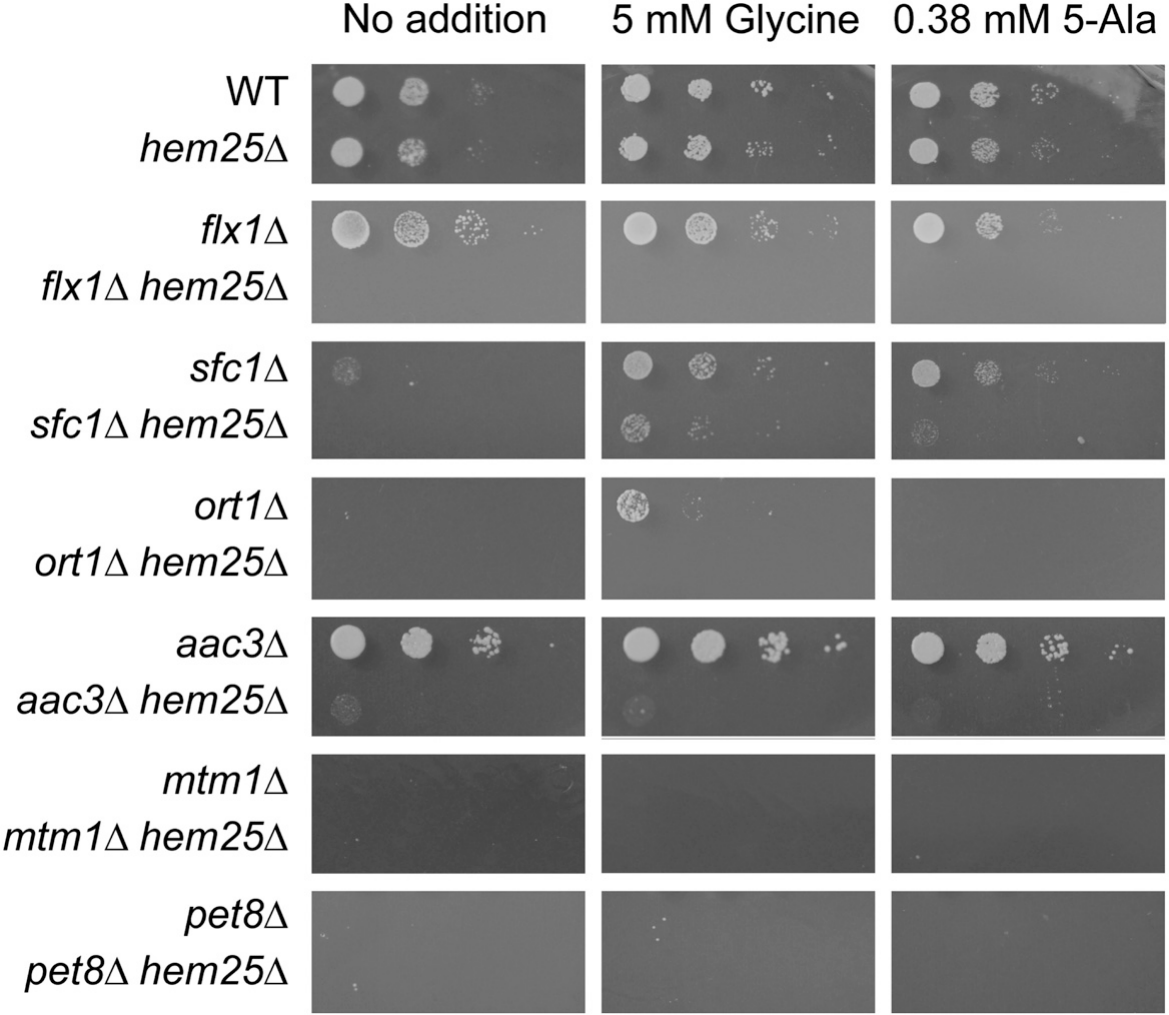
Growth rescue by glycine and 5-Ala on nonfermentable medium for cells lacking specific SLC25 family members in concert with *HEM25*. Yeast strains of the indicated genotypes were grown to early stationary phase in SC-Ura medium supplemented with 5 mM glycine and 0.38 mM 5-Ala to keep the double mutant cells growing without impediment. Cells were washed and resuspended in sterilized water to OD_600 nm_ 0.4, then serially diluted (1:10) and spotted on SD-Ura solid media containing lactate as carbon source. Plates were imaged after 7 d incubation at 30°.

Four out of the six genes whose inactivation decreased cell growth and heme level in concert with loss of Hem25 function are required for growth on nonfermentable carbon sources. These are *SFC1*, *ORT1*, *MTM1*, and *PET8* (Figure 5). An inability to grow on nonfermentable carbon sources generally delineates a poorly functioning ETC. Heme is a component of several components of the ETC (Ajioka *et al.* 2006; Yuan *et al.* 2013).

We determined if the addition of glycine or 5-Ala could enable growth on nonfermentable medium for each of the single mutants identified in our screen, as well as each of these mutants in concert with loss of function of Hem25. Both glycine and 5-Ala enabled growth of *sfc1*Δ strain, while only glycine enabled growth of *ort1*Δ cells. Glycine, and, to a smaller extent, 5-Ala also enabled growth of *sfc1*Δ *hem25*Δ cells. The ability of glycine and 5-Ala to restore growth to *sfc1*Δ cells implies that their inability to grow on a nonfermentable carbon source could be due to their decreased capacity to synthesize heme.

Single mutants *flx1*Δ and *aac3*Δ grew on lactate. Remarkably, the simultaneous loss of Hem25 function completely prevented growth on this nonfermentable carbon source, and it could not be restored by supplementation with glycine or 5-Ala.

### Ablation of HEM25 and FLX1 affects electron transport chain subunit stability

The synthetic growth defect of the *flx1*Δ *hem25*Δ double mutant on a nonfermentable carbon source, but not on a fermentable carbon source, implied the ETC could be compromised. Hem25 imports glycine into the mitochondria, which is required for heme synthesis (Fernández-Murray *et al.* 2016; Lunetti *et al.* 2016). Heme molecules are required to form cytochromes for electron transfer by the ETC (Chiabrando *et al.* 2014; Yuan *et al.* 2013; DiMauro and Schon 2003). Flx1 transports FAD, which is a prosthetic group for flavoproteins, the majority of which are found in the mitochondria where they participate in redox processes of the ETC (Bafunno *et al.* 2004; Giancaspero *et al.* 2014; Spaan *et al.* 2005; Tzagoloff *et al.* 1996; Gudipati *et al.* 2014; Iwata *et al.* 2012; Kim *et al.* 2012; Koch *et al.* 2004). We hypothesized that there would be a decreased stability of FAD and heme containing components of the ETC based on the presence or absence of Flx1 and Hem25. To test this hypothesis, we determined the level of nine different ETC proteins within ETC complexes in wild type, *hem25*Δ, *flx1*Δ, and *flx1*Δ *hem25*Δ strains. The strains were grown in defined media with raffinose as carbon source, and then transferred to lactate medium for 5 hr prior to mitochondria isolation and western blotting to determine the level of each ETC protein examined. Porin (Por1) was used as a load control.

**Figure 6:**
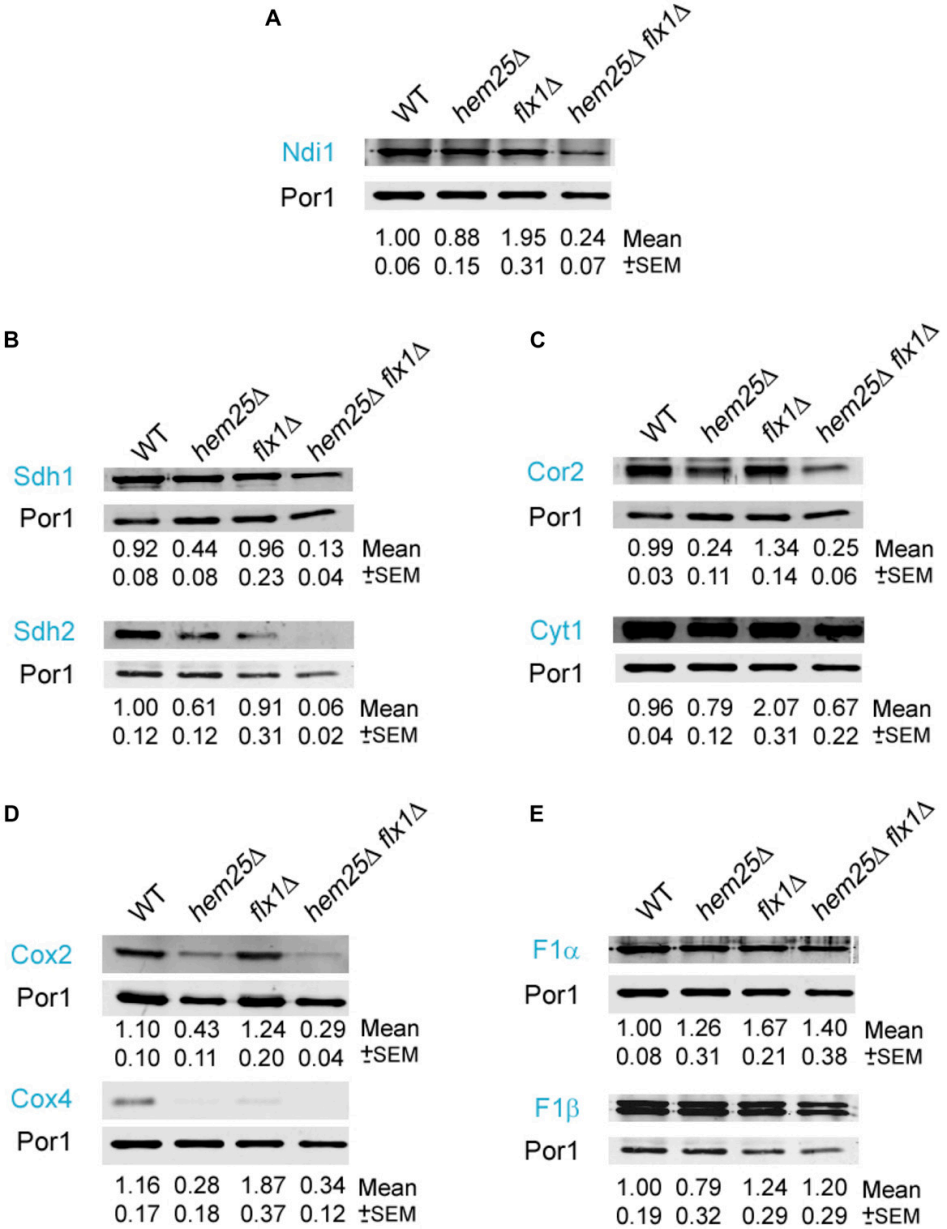
Specific components of the electron transport chain are significantly decreased in *hem25*Δ cells lacking the FAD importer *FLX1*. Cells of the indicated genotypes were grown to an OD of 1.0 in defined medium with raffinose. Cells were then transferred and grown in lactate for 5 hr. Cells were harvested and mitochondrial fractions were prepared and analyzed by western blotting using antibodies specific for (A) Ndi1, (B) Sdh1 and Sdh2, (C) Cor2 and Cyt1, (D) Cox2 and Cox4, and (E) F1α and F1β. Three independent segregant strains of *flx1*Δ and *flx1*Δ *hem25*Δ were used. The figure shown is representative of three independent analyses. Pixel intensity was measured and calculated by using Odyssey Software. Numbers under the lanes represent the mean of protein abundance normalized by the loading control Por1 and then to WT. The mean and the SEM were calculated from three independent western blot analyses. The significance of the differences on protein levels observed between the strains was calculated using ANOVA test with randomized factors (Table S3 in File S1). The deduced protein molecular weight (MW) of the bands revealed by western blotting was consistent with their MW: Ndi1 (57 kDa); Sdh1 (69 kDa) and Sdh2 (30 kDa); Cor2 (40 kDa) and Cyt1 (34 kDa); Cox2 (28 kDa) and Cox4 (17 kDa); F1α (58 kDa) and F1β (55 kDa).

NADH-ubiquinone oxidoreductase, Ndi1, is a component of ETC complex I (Iwata *et al.* 2012). Its level was affected in cells that contain the *flx1*Δ *hem25*Δ double deletion. A significant reduction of Ndi1 levels was observed in cells with the double deletion *flx1*Δ *hem25*Δ compared with the levels observed in the mitochondrial fractions of the single mutant cells (Figure 6A).

Sdh2 and Sdh1 are subunits of succinate dehydrogenase, and form part of complex II of the ETC (Goffrini *et al.* 2009; Kim *et al.* 2012). Cells with the double deletion *flx1*Δ *hem25*Δ showed a decrease in Sdh1 levels compared with the single mutant cells (Figure 6B). A significant decrease in Sdh2 level was also observed in the double mutant in comparison with the single mutants. Sdh1 and Sdh2 levels were also reduced in *hem25*Δ cells (Figure 6B) but not to the extent of that observed in the double mutant cells.

We determined the abundance of subunit 2, Cor2, and cytochrome c1, Cyt1, for complex III of the ETC (di Rago *et al.* 1997; Hunte *et al.* 2000; Schneider and Guarente 1991). Cor2 and Cyt1 levels were significantly decreased in the double mutant *flx1*Δ *hem25*Δ cells (Figure 6C). Both Cor2 and Cyt1 levels were also reduced in *hem25*Δ cells compared with wild-type cells, but not to the extent observed in the double mutants. We also determined the level of the complex IV cytochrome oxidase subunits Cox2 and Cox4 (Ostrander *et al.* 2001; Bottinger *et al.* 2013; Cui *et al.* 2014; Stiburek *et al.* 2012; Su *et al.* 2014). Cox2 and Cox4 were significantly reduced in the *hem25*Δ cells compared with wild-type cells, but there was no reduction in protein levels in the double mutant *flx1*Δ *hem25*Δ compared with the *hem25*Δ cells (Figure 6D). We observed no decrease in F1α and F1β (Su *et al.* 2014) levels for complex V in the double mutant cells in comparison with *flx1*Δ, *hem25*Δ, or wild-type cells (Figure 6E).

There are clear differences in the levels of specific proteins within the ETC upon inactivation of the *HEM25* and *FLX1* genes (Table 2). The complex V subunits F1α and F1β were not affected by loss of function of Hem25, Flx1, or both, while complex IV subunits Cox2 and Cox4 were reduced in cells lacking Hem25 function, but this was not exacerbated by loss of Flx1 function. Interestingly, loss of function of both genes significantly impacted the level of the complex I subunit Ndi1, the complex II subunits Sdh1 and Sdh2, and the complex III subunits Cyt1 and Cor2, compared to loss of either *HEM25* or *FLX1* alone.

**Table 2:** Summary of phenotypes for *HEM25* SLC25 family genetics interactors

	Growth						Heme Level (% WT)		ETC Complex Stability									
Strain	Glycine as N Source	*hem 25*Δ	Dextrose as C Source	*hem 25*Δ	Lactate as C Source	*hem25*Δ	Heme Content	*hem25*Δ	I	*hem25*Δ	II	*hem25*Δ	III	*hem25*Δ	IV	*hem25*Δ	V	*hem25*Δ
Wild type	++++*^a^*		++++		+++		100		+++		+++		+++		+++		+++	
*hem25*Δ	+++		++++		+++		35		+++		++		++		++		+++	
*flx1*Δ	+++	++	++++	++++	—*^b^*	—	49	10	++++	+	+++	+	++++	+	++++	++	+++	+++
*mtm1*Δ	+++	++	++	+*^c^*	—	—	22	19										
*ort1*Δ	+++	+	++++	—	—*^d^*	—	42	5										
*sfc1*Δ	++++	+++	++++	++++	—*^b^*	—*^b^*	40	8										
*pet8*Δ	++	+	++++	++*^b^*	—	—	38	8										
*aac3*Δ	+++++	+++	+++++	++++	++++	—	150	57										

a Relative level.

b Alleviated by glycine and 5-Ala.

c Alleviated by 5-Ala.

d Alleviated by glycine.

**Figure 7:**
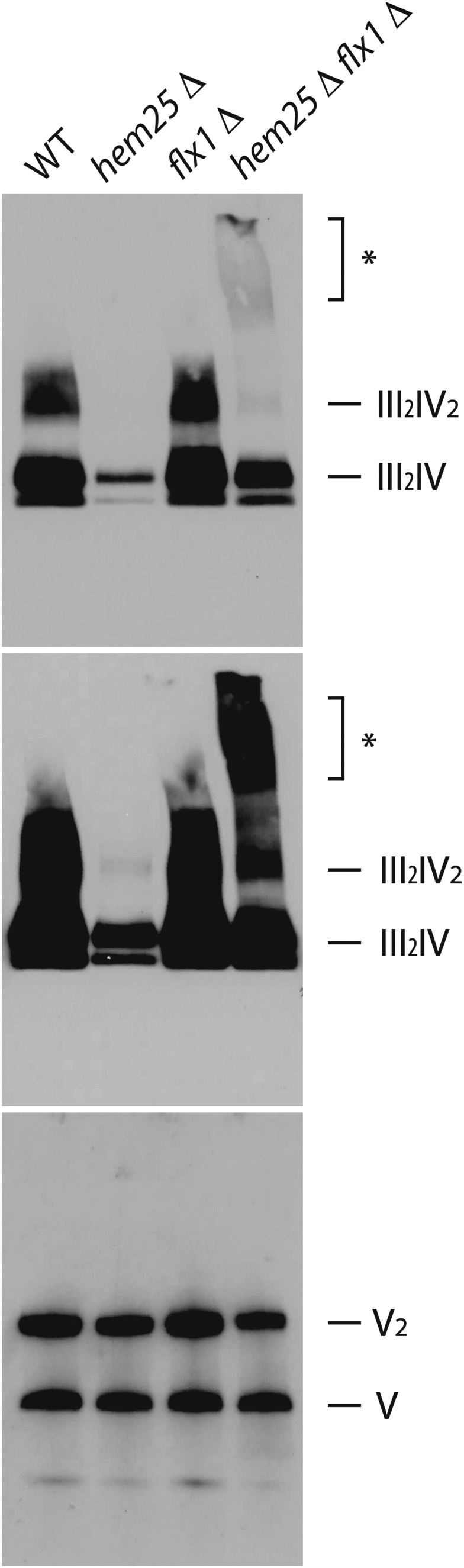
High molecular weight aggregates are present in mitochondria from *flx1*Δ *hem25*Δ cells. Cells of the indicated genotypes were grown to an OD of 1.0 in defined medium with raffinose. Cells were then transferred and grown in lactate for 5 hr. Cells were harvested, mitochondria were isolated and mitochondrial protein complexes were solubilized with digitonin. Protein complexes were resolved by BN-PAGE, transferred into PVDF and analyzed by western blotting using antibodies specific for Cox2 (upper and middle panels; they are identical, middle panel was a longer exposure) or F1α (lower panel). Asterisk denotes the position of high molecular weight aggregates.

To gain a deeper understanding about the inability of double mutant *flx1*Δ *hem25*Δ cells to grow on nonfermentable carbon source, the integrity of the respiratory supercomplexes from digitonin-solubilized mitochondria was assessed by BN-PAGE and western blotting. Consistent with the levels of Cox2 estimated by SDS-PAGE followed by western blotting (see Figure 6D) the status of supercomplexes III_2_IV_2_ and III_2_IV for *flx1*Δ did not differ appreciably compared to wild type cells (Figure 7), whereas, for both *hem25*Δ and *flx1*Δ *hem25*Δ cells a strong reduction of their levels was evident, with supercomplex III_2_IV_2_ barely detectable. Remarkably, very slow mobility forms were observed only in *flx1*Δ *hem25*Δ mitochondrial extracts. The levels of complex V, detected as dimeric and monomeric forms, were not affected by the ablation of *HEM25*, *FLX1*, or both genes, in agreement with data presented in Figure 6E. When mitochondria were solubilized with dodecylmaltoside (Figure S2 in File S1) instead of digitonin, complexes IV and V collapsed into their monomeric forms; however, the Cox2-containing low-mobility forms were still specifically detected in *flx1*Δ *hem25*Δ mitochondrial extracts.

## Discussion

We identified new SLC25 family member genetic interactors with *HEM25*—a mitochondrial SLC25 family member that imports glycine into mitochondria to provide substrate for heme synthesis and the GCV. The genetic interactions between *HEM25* and *FLX1*, *ORT1*, *MTM1*, and *SFC1* or *AAC3* have not been reported, while the genetic interaction of *HEM25* with *PET8* had been reported in the *Saccharomyces* Genome Database (SGD). Of the *HEM25* genetic interactions with SLC25 family members we identified, we studied the genetic interaction between *HEM25* and *FLX1* in depth.

*FLX1* encodes a mitochondrial FAD transporter; the absence of Flx1 affects the import of FAD needed for functional flavoproteins (Bafunno *et al.* 2004; Giancaspero *et al.* 2014; Spaan *et al.* 2005; Tzagoloff *et al.* 1996; Gudipati *et al.* 2014). We found that inactivation of *FLX1* exacerbated the growth defect of *hem25*Δ cells when they were grown using glycine as a sole nitrogen source. Lpd1, which is one of the subunits of the GCV, is a flavoenzyme that uses FAD as a cofactor (Gudipati *et al.* 2014). The reduction of growth on glycine when Flx1 and Hem25 are absent could be due to reduced availability of FAD in the mitochondria, which is required for GCV, and a decrease of glycine inside the mitochondria, which in turn decreases the substrate glycine used by the GCV. Furthermore, when heme content was measured in the single and double mutants, *flx1*Δ *hem25*Δ showed a decrease in heme content compared to either single mutant alone. Protoporphyrinogen oxidase, Hem14, is a mitochondrial flavoprotein (Koch *et al.* 2004) that catalyzes the seventh reaction in the heme synthesis pathway. Thus, the combined defect of Hem25 and Flx1 together could decrease the supply of the initial substrate for heme synthesis, glycine, and also decrease the efficiency of the FAD requiring enzyme Hem14, resulting in a decrease in heme level upon loss of function of both Flx1 and Hem25.

Both heme and FAD are prosthetic groups required to form ETC complexes (Sazanov 2015; Chaban *et al.* 2014). The single mutants *flx1*Δ and *hem25*Δ can proliferate on nonfermentable carbon source, whereas the double mutant could not. Importantly, the decreased ability to incorporate FAD into components of the ETC, together with the strong reduction in heme level observed for *flx1*Δ *hem25*Δ cells, appears to severely compromise the function of the ETC.

Like mammalian cells, the ETC complexes of yeast ETC assemble to form supercomplexes (Cui *et al.* 2014; Pfeiffer *et al.* 2003; Gaspard and McMaster 2015). Our data suggest that Flx1 and Hem25 together are required for stability of specific ETC subunits. The abundance of Ndi1 subunit of complex I decreased in the *flx1*Δ *hem25*Δ cells compared with the levels in the single mutant cells. Complex I is the NADH-ubiquinone oxidoreductase, which has a FAD prosthetic group (Feng *et al.* 2012). The succinate-ubiquinone oxidoreductase is complex II, formed by four subunits, one of which is the flavoprotein Sdh1, which contains FAD bound covalently (Maklashina *et al.* 2016; Kim *et al.* 2012). Sdh2 contains a Fe-S group (Chaban *et al.* 2014; Gudipati *et al.* 2014; Sazanov 2015). The four complex II subunits bind cytochrome *b*, which contains a heme group (Chaban *et al.* 2014; Sazanov 2015; Chiabrando *et al.* 2014). In the western blot analyses, Sdh1 and Sdh2 subunits were significantly decreased in the double mutant cells compared to single mutant cells. The *flx1*Δ *hem25*Δ cells analyzed here also showed a decrease in the abundance of subunit 2 of ubiquinol cytochrome-c reductase (Cor2) and cytochrome c1 (Cyt1) proteins. Cor2 and Cyt1 form part of complex III, and both subunits contain heme molecules (Chaban *et al.* 2014).

Interestingly, we determined that the level of Cox2 and Cox4 proteins from complex IV was decreased in *hem25*Δ cells, but was not further reduced in *flx1*Δ *hem25*Δ cells. This result showed that the absence of Hem25 *per se* affects the stability of Cox4 and Cox2. Cox4 is essential for the assembly and function of the cytochrome *c* oxidase complex (Bottinger *et al.* 2013; Chaban *et al.* 2014). The decrease in heme levels observed in *hem25*Δ cells could affect the expression of Cox4, as its expression is regulated by heme level. If Cox4 is decreased, the Cox2 subunit cannot assemble and is degraded (Nakai *et al.* 1994). A similar situation was observed in yeast cells with a mutation in Cox4, in the absence of Cox4 subunits, Cox2 and Cox3 were not assembled into the higher order complex, and were degraded.

The subunits of the ATP synthase, F1α and F1β, were not diminished in abundance in *flx1*Δ *hem25*Δ cells compared to wild-type cells. The ATP synthase does not contain a heme or FAD molecule in the complex, which might explain why the abundance of these two proteins was not affected. The combined inability to synthesize heme at a wild-type rate and import FAD into the mitochondria results in a decreased ability to assemble ETC complexes that require these cofactors, but does not affect the assembly of ETC complexes where these cofactors are absent. The decreased levels of the ETC proteins studied is likely due to a decrease in the capacity of ETC supercomplexes to properly assemble, due to irregularities in the proper proportions of the proteins within these supercomplexes.

We analyzed the status of Cox2-containing supercomplexes by BN-PAGE. Consistent with the reduced steady state level of Cox2 observed for *hem25*Δ and *flx1*Δ *hem25*Δ cells, these cells showed a strong reduction in supercomplexes formation. Whereas this reduction did not compromise the ability of *hem25*Δ cells to proliferate on nonfermentable sources, *flx1*Δ *hem25*Δ cells did not grow on lactate. We detected, in mitochondria isolated from *flx1*Δ *hem25*Δ cells, the presence of uncharacterized Cox2-containing high molecular weight species. We propose that the simultaneous deficiency of FAD and heme could prompt the accumulation of precursors of the ETC in their apo-forms, leading to the aggregation of improper folded molecules, and saturation of the proteolytic capacity of the mitochondria. These combined effects would prevent the normal assemble and function of the ETC, thus impairing cell respiration.

Beyond *FLX1*, several other novel genetic interactions between *HEM25* and other SLC25 family members were observed. *MTM1* encodes a putative mitochondrial high affinity PLP transporter (Whittaker *et al.* 2015). PLP is a cofactor required by Lpd1, which is one of the subunits of GCV. The growth defect of *mtm1*Δ *hem25*Δ cells using glycine as the sole nitrogen source was not significantly different from *mtm1*Δ cells, and it could be due to reduction of PLP levels in the mitochondria. This reduction would affect GCV function. A reduction of mitochondrial glycine levels upon *HEM25* inactivation would barely impact growth, as Hem25 lies upstream of GCV. When heme was measured, a decrease in heme content was observed in *mtm1*Δ cells, but the double mutant *hem25*Δ *mtm1*Δ cells had equal heme levels to the *mtm1*Δ cells. PLP is an essential cofactor of Hem1 (Astner *et al.* 2005), which is the first enzyme in the heme synthesis pathway (Ajioka *et al.* 2006; Chiabrando *et al.* 2014). The absence of Mtm1 would be expected to impact the function of Hem1 due to the decreased availability of the cofactor PLP. The double mutant cells probably did not have a heme level significantly lower than *mtm1*Δ cells, because, under the situation where Hem1 function is impaired by decreased PLP levels, the availability of glycine would not determine the rate of heme synthesis, as Hem25 lies upstream of Hem1 in the heme biosynthetic pathway. In contrast, *mtm1*Δ *hem25*Δ cells also showed a growth defect relative to single mutants when cells grew in dextrose medium without supplementation. The growth phenotype of the double mutant cells was improved by the addition of 5-Ala. 5-Ala is a downstream metabolite of Hem1, the first enzymatic reaction for heme synthesis, and it would bypass the deficiency of PLP and glycine. A decreased ability to import PLP clearly affects growth of cells when mitochondrial glycine import is also decreased. This appears to be due to the fact that glycine is a substrate for the GCV and for heme synthesis, and both pathways contain PLP-dependent enzymes. Simultaneous restriction of both GCV and heme synthesis is a likely explanation for the decreased growth of cells lacking Hem25 and Mtm1 function.

*SFC1* encodes a mitochondrial succinate-fumarate transporter, which transports succinate into, and fumarate out of, mitochondria (Lin *et al.* 2011). When heme content was measured in cells lacking Sfc1 and Hem25, a severe decrease in heme content was observed compared to *sfc1*Δ or *hem25*Δ cells. The import of succinate and export of fumarate would increase the availability of succinate in the mitochondria for synthesis of succinyl-CoA by the tricarboxylic acid (TCA) cycle. Succinyl-CoA and glycine are the substrates for the first reaction of heme synthesis (Ajioka *et al.* 2006; Chiabrando *et al.* 2014). A deletion of *SFC1* and *HEM25* would deprive the mitochondria of the two substrates required for heme synthesis. Our phenotypic analysis of growth in lactate showed that the decrease in growth observed in *sfc1*Δ *hem25*Δ cells was partially restored by the addition of 5-Ala or glycine. This result could indicate that decreased succinyl-CoA and glycine limit heme synthesis, and possibly other mitochondrial processes in addition to the synthesis of heme.

*ORT1* encodes an ornithine transporter of the mitochondrial inner membrane, which exports ornithine from mitochondria in exchange for protons (Marobbio *et al.* 2015). *ORT1* had a strong genetic interaction with *HEM25*. When *ort1*Δ *hem25*Δ cells were grown in glycine as sole nitrogen source, there was a synthetic growth impairment compared to the respective single mutants. In growth on dextrose, the double mutant cells also showed a strong growth impairment. When heme content was measured, the double mutant *ort1*Δ *hem25*Δ cells had a severe decrease in heme levels that exceeded the mere cumulative effect of both single mutations. The *ORT1* gene has been reported to genetically interact with *PET8* (Szappanos *et al.* 2011). *PET8* encodes a mitochondrial S-adenosylmethionine (SAM) transporter (Marobbio *et al.* 2003), and *pet8*Δ cells had their heme levels reduced by 50%. The *pet8*Δ *hem25*Δ cells showed a further decrease in heme content compared with their respective single mutants. There is a clear genetic link between *ORT1*, *PET8*, and *HEM25* that revolved around the role of heme in mitochondrial function; however, more work will be required to ascertain the biochemical underpinnings of these genetic interactions.

Our phenotypic analysis also showed that in nonfermentable media, *aac3*Δ *hem25*Δ cells had a severe growth defect. *AAC3* encodes a mitochondrial ADP/ATP translocator, which exchanges cytosolic ADP for mitochondrially synthesized ATP (Kolarov *et al.* 1990; Smith and Thorsness 2008). It has a role in maintenance of viability in respiratory conditions. However, during exponential growth on dextrose under aerobic conditions, it acts in the opposite direction, importing ATP into mitochondria (Azuma *et al.* 2008). Disruption of the *AAC* genes in yeast had previously been determined to result in a reduction of heme biosynthesis by blocking the translocation of heme precursors into the matrix. Although the reason why a reduction in growth is observed when Hem25 and Aac3 are absent in the cells is not known, a possible explanation of for the growth defect is that heme synthesis could be impaired (Azuma *et al.* 2008). The function of *AAC3* has to be further studied to understand why it has a genetic interaction with *HEM25*.

Defects in heme synthesis can lead to various disease states in humans. Congenital sideroblastic anemias are caused by defects in the early steps in heme synthesis, including those encoding the human homologs of yeast *HEM25* (*SLC25A38* in humans) and *HEM1* (*ALAS2* in humans) (Aivado *et al.* 2006; Boycott *et al.* 2013, 2014; Guernsey *et al.* 2009; Aoki *et al.* 1973; Cotter *et al.* 1992). It could be interesting to determine if defects (polymorphisms or mutations) in any of the genes identified here that affect the growth, level of heme, and ETC stability in yeast cells lacking Hem25 function, modify the phenotype of congenital sideroblastic anemia patients.

## Supplementary Material

Supplemental material is available online at http://www.g3journal.org/lookup/suppl/doi:10.1534/g3.117.041194/-/DC1.
