# Supplementary material for: SLC25 Family Member Genetic Interactions Identify a Role for HEM25 in Yeast Electron Transport Chain Stability
Source: G3 (Bethesda). 2017 Apr 12;7(6):1861–73. doi: 10.1534/g3.117.041194 (PMC5473764; doi:10.1534/g3.117.041194)
Supplement: Supplementary file 1 [file 1861FileS1.docx]

**Supplemental Tables**

**Table S1. Yeast strains used in this study**

| **Strain*** | **Genotype** |
| --- | --- |
| Y2454 | MATα *mfa1*Δ*::MFA1pr-HIS3 can1*Δ*0 his3*Δ*1,15 leu2*Δ*0 ura3*Δ*0 lys2*Δ*0* |
| NDY 005 | Y2454 *hem25*Δ*::NatMX4* |
| BY 4741 | MAT**a***his3*Δ*1 leu2*Δ*0 met15*Δ*0 ura3*Δ*0* |
| NDY 009 | MATα *his3*Δ*1 leu2*Δ*0 met15*Δ*0 ura3*Δ*0 hem25*Δ*::KanMX4* |
| NDY 297 | MAT**a***leu2*Δ*0 ura3*Δ*0 can1*Δ*0 hem25*Δ*::NatMX4* |
| NDY 487 | MAT**a***leu2Δ0 ura3*Δ*0 can1*Δ*0 aac3*Δ*::KanMX4* |
| NDY 492 | MAT**a***leu2Δ0 ura3*Δ*0 can1*Δ*0 mtm1*Δ*::KanMX4* |
| NDY 485 | MAT**a** *leu2Δ0 ura3*Δ*0 can1*Δ*0 sfc1*Δ*::KanMX4* |
| NDY 486 | MAT**a***leu2Δ0 ura3*Δ*0 can1*Δ*0 pet8*Δ*::KanMX4* |
| NDY 489 | MAT**a***leu2Δ0 ura3*Δ*0 can1*Δ*0 ort1*Δ*::KanMX4* |
| NDY 338 | MAT**a** *leu2Δ0 ura3*Δ*0 can1*Δ*0 lpd1*Δ*::KanMX4* |
| NDY 210 | MAT**a** *leu2Δ0 ura3*Δ*0 can1*Δ*0 aac3*Δ*::KanMX4 hem25*Δ*::NatMX4* |
| NDY 230 | MAT**a** *leu2Δ0 ura3*Δ*0 can1*Δ*0 mtm1*Δ*::KanMX4 hem25*Δ*::NatMX4* |
| NDY 207 | MAT**a** *leu2Δ0 ura3*Δ*0 can1*Δ*0 sfc1*Δ*::KanMX4 hem25*Δ*::NatMX4* |
| NDY 224 | MAT**a** *leu2Δ0 ura3*Δ*0 can1*Δ*0 pet8*Δ*::KanMX4 hem25*Δ*::NatMX4* |
| NDY 226 | MAT**a** *leu2Δ0 ura3*Δ*0 can1*Δ*0 ort1*Δ*::KanMX4 hem25*Δ*::NatMX4* |
| NDY 586 | MAT**a***leu2Δ0 ura3*Δ*0 can1*Δ*0 flx1*Δ*::KanMX4 hem25*Δ*::NatMX4* |

* Double mutant strains were generated by mating of the single mutants, diploid selection, sporulation and selection of haploids cells. At least three independent double mutant segregants were isolated from each genetic cross.

**Table S2. Antibodies used in this study**

| **Primary antibodies** | **Dilution** | **Source** |  |  |
| --- | --- | --- | --- | --- |
| anti cytochrome c oxidase, subunit 2 (Cox2) | 1 1,000 | Invitrogen (Mitosciences, Inc) |  |  |
| anti cytochrome c oxidase, subunit 4 (Cox4) | 1 2,000 | Abcam |  |  |
| anti cytochrome c1 (Cyt1) | 1 1,000 | received as a gift from Dr. Carla M. Koehler | | |
| anti ubiquinol cytochrome-c reductase, subunit 2 (Cor2) | 1 1,000 | received as a gift from Dr. Carla M. Koehler | | |
| anti complex V (F1*α*) | 1 1,000 | received as a gift from Dr. Carla M. Koehler | | |
| anti complex V (F1*β*) | 1 1,000 | received as a gift from Dr. Carla M. Koehler | | |
| anti succinate dehydrogenase, subunit 1 (Sdh1) | 1 5,000 | received as a gift from Dr. Dennis R. Winge | | |
| anti succinate dehydrogenase, subunit 2 (Sdh2) | 1 5,000 | received as a gift from Dr. Dennis R. Winge | | |
| anti NADH:ubiquinone oxidoreductase (Ndi1) | 1 125 | received as a gift from Dr. Takao Yagi | |  |
| anti mitochondrial porin (Por1) | 1 2,000 | Abcam |  |  |
| **Secondary antidodies** | **Dilution** | **Source** |  |  |
| HRP-Goat anti-mouse | 1 3,000 | Cell Signalling |  |  |
| HRP Goat anti-rabbit | 1 3,000 | Cell Signalling |  |  |
| Goat anti-mouse | 1 15,000 | LI-COR |  |  |
| Goat anti-rabbit | 1 5,000 | LI-COR |  |  |

**Table S3**

Significance for the differences between the indicated strains in the expression levels of protein components of the ETC quantified from Figure 6^a^

| Proteins | *hem25*Δ: *flx1*Δ *hem25*Δ | *flx1*Δ: *flx1*Δ *hem25*Δ | WT: *hem25*Δ | WT: *flx1*Δ |
| --- | --- | --- | --- | --- |
| Ndi1 | 0.0088 | 0.0016 | 0.5007 | 0.0485 |
| Sdh1 | 0.0117 | 0.0125 | 0.0143 | 0.8874 |
| Sdh2 | 0.0033 | 0.0320 | 0.0858 | 0.8353 |
| Cor2 | 0.9261 | 0.0001 | 0.0002 | 0.0527 |
| Cyt1 | 0.0607 | 0.0062 | 0.2257 | 0.0160 |
| Cox2 | 0.5160 | 0.0034 | 0.0319 | 0.4407 |
| Cox4 | 0.6007 | 0.0022 | 0.0126 | 0.0146 |
| F1α | 0.7975 | 0.5573 | 0.4591 | 0.0472 |
| F1β | 0.3947 | 0.9350 | 0.6189 | 0.5537 |

^a^Differences between strains were determined using ANOVA test with randomized factors. At least three independent experiments were analyzed to calculate the p values.

**Supplemental Figures**

**
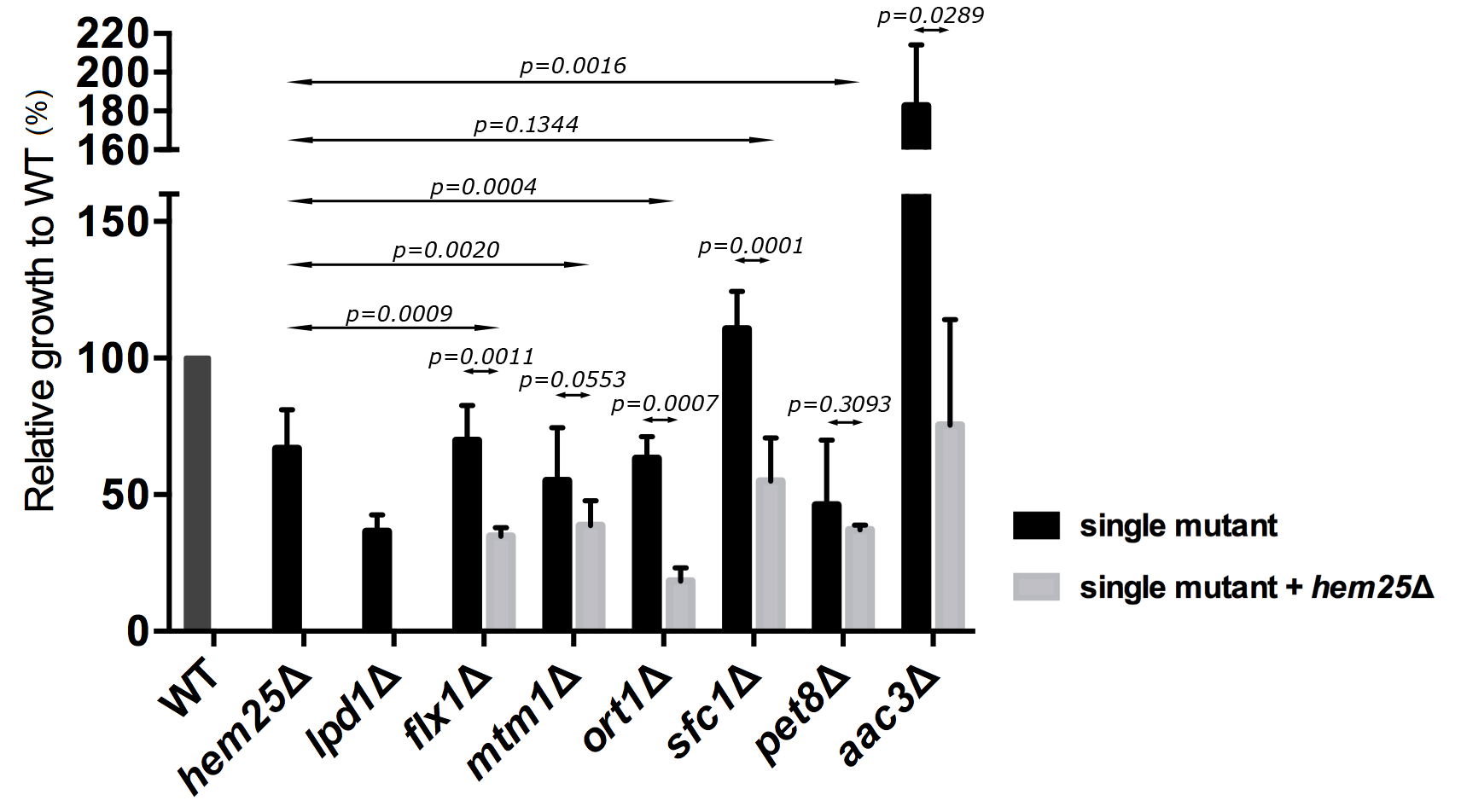
A**

**B
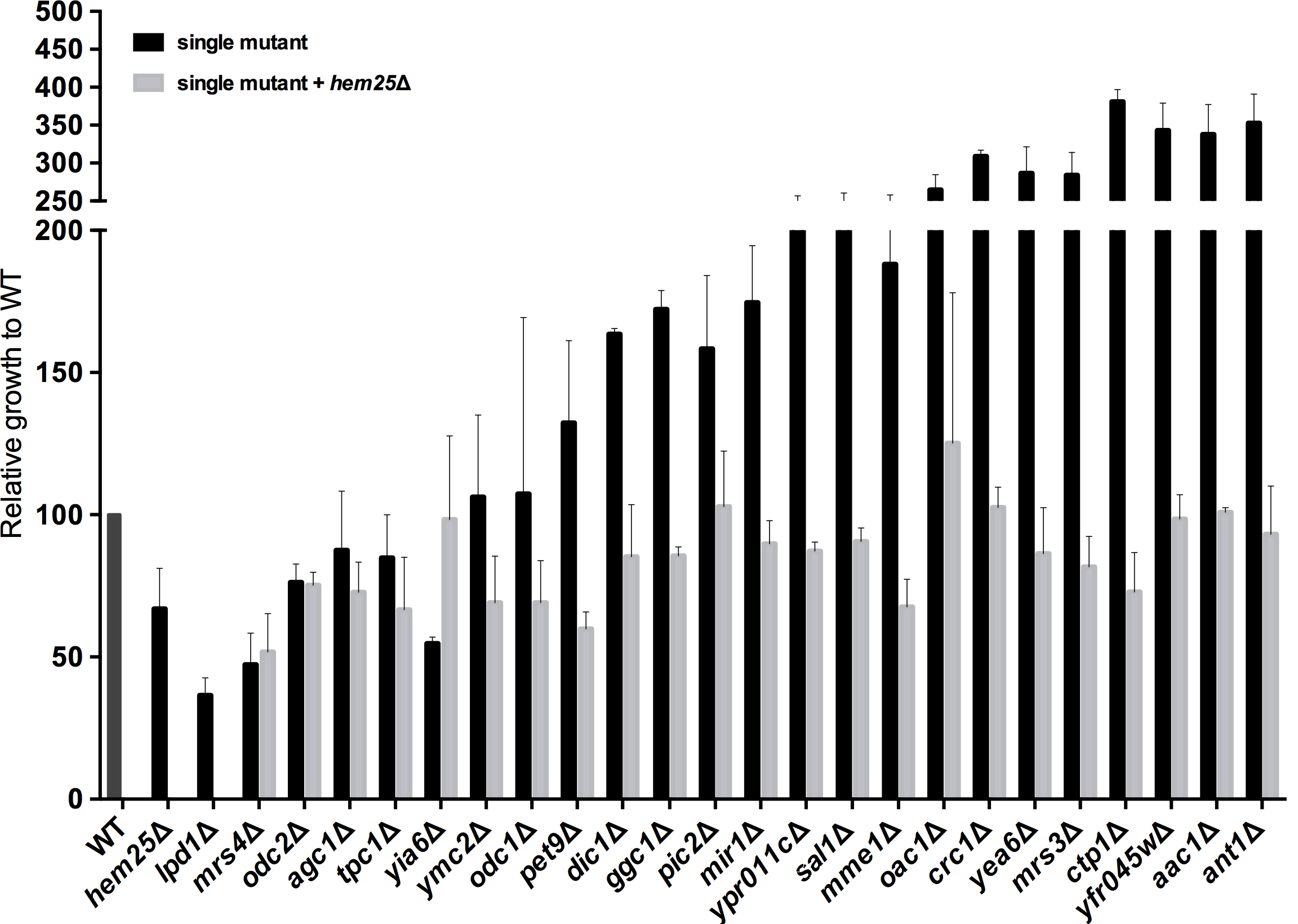
**

**Figure S1**. Growth analysis on glycine as sole nitrogen source for yeast strain with deleted genes for 29 members of the SLC25 family along with *HEM25*. Cells of the indicated genotypes were grown in SD-Ura medium using 1 g/L of ammonium sulfate, washed and inoculated at OD600nm 0.1 in SD-Ura medium with 30 g/L glycine as sole nitrogen source. Cells were cultured at 30°C. OD was measured after 5 days of growth. The numbers represented in the graph are the percentages relative to wild type (WT). WT growth was OD600nm of 1.05 ± 0.05 (SEM). At least three independent experiments were done to calculate the mean and standard deviation. **Panel A**. Selected six members of the SLC25 family. Differences between single deletion strains and double deletion strains were determined using a fixed factor ANOVA test. At least three independent experiments were done to calculate the p values. **Panel B**. Remaining 23 members of the SLC25 family.

**Figure S2**. Blue Native PAGE analysis of dodecylmaltoside solubilized mitochondria. Cells of the indicated genotypes were grown to an OD of 1.0 in defined media with raffinose. Cells were then transferred and grown in lactate for 5 hrs. Cells were harvested, mitochondria were isolated and mitochondrial protein complexes were solubilized with dodecylmaltoside. Protein complexes were resolved by BN-PAGE, transferred into PVDF and analyzed by western blotting using antibodies specific for Cox2 (upper and middle panels; they are identical, middle panel was a longer exposure) or F1α (lower panel). Asterisk denotes the position of high molecular weight aggregates.
